# Scoping review of digital technologies for oral health education among adolescents in sub-Saharan Africa

**DOI:** 10.4102/jphia.v16i4.1509

**Published:** 2025-12-04

**Authors:** Richard O. Oveh, Omorinola A. Afolabi, Anita Dabar, Maha El Tantawi, Moréniké O. Foláyan

**Affiliations:** 1 Department of Information and Communication Technology, Faculty of Computing, University of Delta, Agbor, Nigeria; 2 Department of Oral Health Initiative, Nigerian Institute of Medical Research, Lagos, Nigeria; 3 Department of Social Development, Moshood Abiola Polytechnic, Abeokuta, Nigeria; 4 Department of Public Health, Lead City University, Ibadan, Oyo State, Nigeria; 5 Department of Paediatric Dentistry and Dental Public Health, Faculty of Dentistry, Alexandria University, Alexandria, Egypt; 6 Department of Child Dental Health, Obafemi Awolowo University, Ile-Ife, Osun State, Nigeria

**Keywords:** digital technology, oral health, education, adolescents, young adults, sub-Saharan Africa

## Abstract

**Background:**

There is a scarcity of knowledge on digital technology utilisation for oral health education in sub-Saharan Africa for adolescents and young adults.

**Aim:**

This study assessed the scope of digital technology utilisation for oral health education for adolescents and young adults in sub-Saharan Africa.

**Setting:**

The review focused on studies conducted within sub-Saharan African countries and aimed at adolescents and young adults aged 10–19 years.

**Methods:**

The scoping review was registered in the Open Science Framework. A systematic search of the literature was conducted in May 2024 and updated in August 2025 on PubMed, Scopus, Web of Science, ACM and Google Scholar. The search terms included the phrases ‘oral health education’, ‘digital technology’, ‘adolescents’, ‘young adults’ and ‘sub-Saharan Africa’. The extracted details were guided by the World Health Organization framework for digital health interventions. Two independent reviewers conducted abstract and title screening, full-text screening and data extraction following the inclusion and exclusion criteria. One reviewer handled conflict resolution.

**Results:**

A total of 3745 records were identified, of which two studies met the inclusion criteria. The studies were conducted in Nigeria between 2013 and 2025. They were community-based, and Telehealth was used for health awareness broadcasts and education.

**Conclusion:**

The utilisation of digital technology in oral health education in sub-Saharan Africa is still low.

**Contribution:**

This study highlights the effectiveness of culturally adapted, video-based interventions delivered in indigenous languages for improving oral health education among adolescents in sub-Saharan Africa.

## Introduction

In sub-Saharan Africa, non-communicable diseases, including oral health conditions, are posing increasing challenges to health systems.^1,2^ The region has the steepest global rise in oral health diseases in the last three decades.^3^ Adolescents in the region also have high unmet dental needs.^4^ As poor oral health is associated with pain and discomfort, tooth loss, impaired oral functioning, deformity and missed school time, adolescents who have poor oral health have a lower quality of life.^5^

Oral diseases are preventable. Increasing oral health awareness through health education can improve oral health among adolescents.^6,7,8,9^ The methods of health education, however, influence oral health knowledge acquisition among adolescents.^10^ The use of aids such as lectures, models, albums, e-programme, games and drawings for children can improve oral status and health knowledge.^11^

The traditional methods of oral health education fail to reach and engage adolescents and young adults effectively, making them vulnerable to preventable dental problems.^12^ Alternative approaches to oral health education could be through the use of digital technology, as it breaks the barriers of distance and time and boosts engagement.^13^ Digital tools are cost-effective for promoting healthy behaviours at scale.^13^

Digital technology presents a promising opportunity for delivering oral health education to adolescents in sub-Saharan Africa.^14^ This is because it has gained acceptance and adoption among adolescents and young adults for various purposes^15^ and has the potential to ensure quality oral health education in line with the United Nations Sustainable Development 2030 Goal 4 (SDG 4), which focuses on ensuring inclusive education for everyone.^16^ Understanding how digital technology can be used effectively to address the oral health knowledge gap among adolescents can help address the observed growing gap in oral health in the region. This scoping review assesses the scope of digital technology utilisation for oral health education among adolescents and young adults in sub-Saharan Africa. The objectives of the review were (1) to identify and catalogue studies, programmes and initiatives utilising digital technology for oral health education among adolescents and young adults in sub-Saharan Africa and (2) to analyse the characteristics, methodologies and outcomes of identified interventions.

## Research methods and design

This scoping review was registered with the Open Science Framework (OSF) Registries. The Arksey and O’Malley framework^17^ was used as the guide for conducting this scoping review. It consists of five main steps: (1) formulating the research questions, (2) identifying relevant studies, (3) selecting the studies, organising the data, and (4) compiling, summarising, and (5) presenting the findings.

### Identifying the research question

The research question of the scoping review was: How are digital interventions utilised for oral health education among adolescents in sub-Saharan Africa? The population, concept and context (PCC) framework^18^ was used for this review. The population was adolescents, the concept was digital technology for oral health education, and the context was sub-Saharan Africa.

### Identification of relevant studies

A search strategy was developed to identify relevant studies. The search was conducted in PubMed, Web of Science, Scopus, Association for Computing Machinery (ACM) Digital Library and Google Scholar using prespecified search terms. Titles, abstracts or keywords were used to identify the search queries. Mapping terms to subject headings (i.e. MeSH terms) was employed in relevant databases to enhance the efficiency and accuracy of the search. The search terms included the phrases ‘oral health education’, ‘digital technology’, ‘adolescents’, ‘young adults’ and ‘sub-Saharan Africa’. Details of the search strategies used for each database are in Appendix 1, Table 1-A1.

### Study selection

Publications and grey literature identified through the search strategy were downloaded, imported into Covidence, and duplicates were removed. Two independent reviewers (Z.C. and A.D.) screened the titles and abstracts of identified records against the exclusion and eligibility criteria. When required, two independent reviewers retrieved and assessed full-text articles of relevant studies for eligibility. Conflicts during the review were resolved through discussion for consensus. The studies were eligible if they included adolescents aged 10–19 years^19^ or if data could be independently reported for adolescents within the age range; if the study setting was in sub-Saharan Africa; if the study addressed oral health education using digital technology; and if full texts were available. The exclusion criteria included studies not about oral health education, studies using other technologies like pamphlets, posters or physical oral health education, studies where the description of digital technology could not be extracted, and studies not published in English.

### Data charting

An Excel sheet was developed to chart the data extracted from the included resources. The sheet was checked to assess its comprehensiveness and alignment with the study purpose. The extracted items included the type of resource (paper, newspaper article, website, social media), year of publishing or establishment, country of deployment, type of agency developing the digital intervention (public or private), level of deployment (local, national, regional), language, purpose of digital health intervention (DHI), type of DHI service or application, number of users and age of targeted users.

### Collating, summarising and reporting results

The extracted data were compiled and displayed in a table. A narrative summary was then employed to present an overview of the identified evidence.

### Ethical considerations

This article followed all ethical standards for research without direct contact with human or animal subjects.

## Results

**FIGURE 1:**
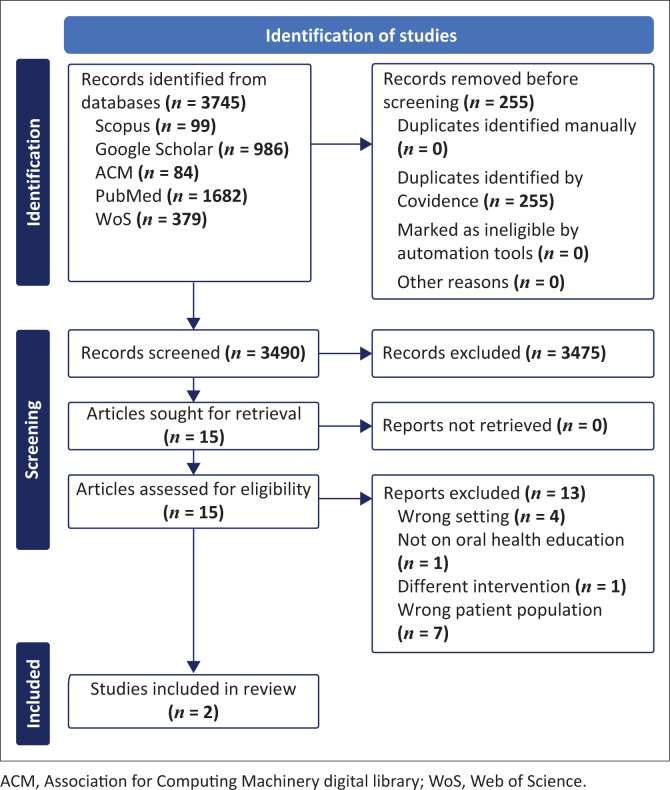
Preferred Reporting Items for Systematic Reviews and Meta-Analysis (PRISMA) flow diagram of the study.

The search yielded 3745 records, as shown in Figure 1. Once these records were compiled, 255 duplicate records were removed before screening, leaving 1898 records for further review. At the title and abstract screening stage, 3475 records were excluded. A total of 15 studies were sought for retrieval, of which 13 were excluded for the following reasons: wrong setting (*n* = 4), not on oral health education (wrong study design) (*n* = 1), non-digital intervention (*n* = 1) and wrong patient population (*n* = 7). Overall, two^20,21^ studies were included in the review.

### Study characteristics

Table 1 shows the population characteristics with two included studies.^20,21^ The first study was targeted at adolescents aged between 11 years and 12 years. The study was conducted in Nigeria^20^ in 2013. The interventions had public ownership. It was conducted in a city (Ibadan) in the Yoruba language. The purpose of intervention was oral hygiene and health awareness through broadcasts. The type of digital health services was community-based information.^20^ The study was a randomised controlled trial to evaluate the efficacy of video as a dental health teaching instrument. A total of 120 children between the ages of 11 years and 12 years, who were chosen at random from three Nigerian public elementary schools in Ibadan, participated in an interventional study. Three study groups were created: the first group saw the film, the second group heard an oral elucidation of dental health in Yoruba, and the third group served as the control. After receiving a complete mouth prophylaxis, each participant’s oral hygiene was evaluated using Greene and Vermillion’s Streamlined Oral Hygiene Index 6 weeks later. The oral hygiene of individuals in the verbal instruction group improved by 23.4% when compared to the control group, while participants in the video group improved by 28.6%. The study found that a culturally appropriate video presented in an indigenous language significantly improved oral hygiene among school children from lower socioeconomic backgrounds. Cultural inclusion increased the acceptance and use of DHI, as it gives a sense of belonging. Additionally, the study highlighted a gap in research regarding the effectiveness of verbal oral health education delivered in local Nigerian languages.

**TABLE 1:** Characteristics of the studies included in the scoping review.

Serial number	Type of resource	First author	Country of deployment	Agency developing the digital intervention	Level of deployment	Language	Purpose of DHI	Type of DHI service or application	Age of targeted users (years)
1	Article	Olubunmi^20^	Nigeria	Public	Local	Yoruba	Health awareness broadcast	Community-based information system	11–12
2	Article	Lawal^21^	Nigeria	Private	Local	English, Pidgin English and Yoruba	Health education	Telehealth	10–19

Note: Please see the full reference list of the article, Oveh RO, Afolabi OA, Dabar A, Tantawi ME, Foláyan MO. Scoping review of digital technologies for oral health education among adolescents in sub-Saharan Africa. J Public Health Africa. 2025;16(4), a1509. https://doi.org/10.4102/jphia.v16i4.1509, for more information.

DHI, digital health intervention

The second study^21^ was conducted in 2025 and targeted adolescents aged 10–19 years. The study presents the creation and validation of animated videos designed as an oral health training resource for adolescents and educators in Ibadan, Nigeria. It involved a focus group discussion (FGD) with 201 adolescents, which explored their views on the use of video as a training tool for oral health promotion. The narrative video was created by a multidisciplinary team comprising public health dentists, paediatric dentists, epidemiologists, communication scientists, animation specialists, dental surgery residents, a paediatric surgeon and science educators. The development process encompassed pre-video production research, formulation of key oral health education messages, script writing and storyboarding, character creation and design, visual style determination, voice-over narration recording, production and post-production processing. The video script was created using material derived from a guidebook on student dental healthcare. The video was assessed for face and content quality by dentists, educators and secondary school students via individual comments and FGDs. The results showed strong enthusiasm among adolescents for using video as a pedagogical instrument and a desire for its content to encompass information pertaining to the oral diseases as well as the methodologies for dental care. The adolescents expressed their approval of the video. It was determined to be user-friendly, plainly illustrated, and exceptionally informative. The drawback of this study is that the validation process relied solely on qualitative methods, without quantitative input that could have facilitated response quantification.

## Discussion

Both studies focused on improving oral health awareness and practices among Nigerian adolescents, specifically within Ibadan, through DHIs that were culturally appropriate and delivered in the local Yoruba language. Despite being conducted more than a decade apart (2013 vs. 2025), the studies collectively highlight the progressive role of digital media – particularly video – in adolescent oral health education.

The first study^20^ employed a randomised controlled trial design involving 120 adolescents aged 11–12 years across three public schools. It evaluated the effectiveness of videos versus verbal instruction (both in Yoruba) in promoting oral hygiene, with a control group for comparison. The findings revealed that both video and verbal interventions improved oral hygiene compared to the control group, but the video intervention was more effective, resulting in a 28.6% improvement in oral hygiene compared to 23.4% for the verbal instruction group. Importantly, the study underscored the value of cultural inclusion by demonstrating that interventions presented in indigenous languages foster greater engagement and effectiveness among children from lower socioeconomic backgrounds. However, while the study captured measurable improvements in oral hygiene, it revealed a gap in the research – namely, the need to evaluate verbal oral health education in local languages more systematically, as its impact, though positive, was less well-studied.

The second study^21^ expanded upon the 2013 work by not just evaluating but also co-creating a culturally relevant, animated video resource for oral health education targeted at adolescents aged 10–19 years. Unlike the first study, which measured direct health outcomes (oral hygiene improvements), the second study emphasised participatory design, involving 201 adolescents in FGDs to validate the relevance, clarity and acceptability of the intervention. Adolescents demonstrated strong enthusiasm for the video and valued its simple illustrations, narrative style and educational content. The participatory process enhanced the video’s cultural and contextual resonance, while the involvement of a multidisciplinary team ensured technical accuracy and pedagogical soundness. Nonetheless, a limitation was identified – the validation process relied exclusively on qualitative feedback, lacking quantitative assessments that could measure behavioural or health-related impact, such as improvements in oral hygiene.

The studies reveal an evolving approach to oral health promotion: the first provided evidence of video effectiveness in improving oral hygiene outcomes, while the second advanced the process by prioritising co-creation and participatory validation of educational tools. Both highlight that cultural tailoring (especially through indigenous language and contextually relevant narratives) enhances adolescents’ acceptance and learning. This study has several limitations that must be acknowledged. Only two eligible studies were identified, both conducted in Ibadan, Nigeria, which restricts the breadth of evidence and limits the generalisability of findings across sub-Saharan Africa. The focus on a single geographic and cultural setting does not reflect the diversity of oral health challenges, languages and digital ecosystems in the wider region.

## Conclusion

The two studies demonstrate the potential of culturally appropriate, video-based DHIs to improve oral health education among adolescents in Nigeria. The 2013 study provided quantitative evidence that video interventions outperform verbal instructions in enhancing oral hygiene, while the 2025 study reinforced the importance of participatory design and cultural contextualisation in ensuring relevance and adolescent acceptance. Collectively, they show that digital health innovations, when rooted in local language and culture, can bridge gaps in oral health education, especially for adolescents in underserved communities. However, future research should integrate both qualitative and quantitative approaches, thereby not only validating acceptability but also measuring tangible health outcomes.

## Appendix 1

**TABLE 1-A1:** Search strategy.

Serial number	Database	Search string
1	PubMed	(‘cell phone’ OR ‘mobile phone’ OR telephone OR ‘digital phone’ OR ‘mobile app’ OR ‘mobile application’ OR digital OR electronic OR automatic OR cyber OR automated OR programmed OR online OR multimedia OR ‘web-based’ OR robotic OR interactive OR virtual OR ‘digital technology’ OR ‘information technology’ OR technology OR tech OR ‘digital technologies’ OR e-health OR eHealth OR m-health OR mHealth OR ‘mobile health’ OR ‘mobile health applications’ OR ‘mobile apps’ OR ‘health apps’ OR ‘health applications’ OR ‘health tech’ OR telehealth OR telemedicine OR telecare OR ‘online health’ OR ‘web-based health’ OR ‘internet-based health’ OR ‘digital tools’ OR ‘digital platforms’ OR ‘digital solutions’ OR ‘electronic health’ OR ‘electronic health records’ OR ‘smartphone health’ OR ‘smartphone apps’ OR ‘wearable technology’ OR wearables OR ‘virtual health’ OR ‘virtual care’ OR ‘big data’ OR ‘artificial intelligence’ OR ‘real-time’ OR video OR ‘Digital Technology’[Mesh] OR ‘Information Technology’[Mesh] OR ‘Digital Health’[Mesh] OR ‘mobile oral health’ OR applications OR apps OR ‘smart phone’) AND (oral health OR dental care OR dental health OR dental hygiene OR oral care OR odontic) AND (Angola OR Benin OR Botswana OR ‘Burkina Faso’ OR Burundi OR Cameroon OR ‘Cape Verde’ OR ‘Central African Republic’ OR Chad OR Comoros OR ‘Congo (Brazzaville)’ OR ‘Congo (Democratic Republic)’ OR ‘Côte d’Ivoire’ OR Djibouti OR ‘Equatorial Guinea’ OR Eritrea OR Ethiopia OR Gabon OR ‘The Gambia’ OR Ghana OR Guinea OR ‘Guinea-Bissau’ OR Kenya OR Lesotho OR Liberia OR Madagascar OR Malawi OR Mali OR Mauritania OR Mauritius OR Mozambique OR Namibia OR Niger OR Nigeria OR Réunion OR Rwanda OR ‘Sao Tome and Principe’ OR Senegal OR Seychelles OR ‘Sierra Leone’ OR Somalia OR ‘South Africa’ OR Sudan OR Swaziland OR Tanzania OR Togo OR Uganda OR ‘Western Sahara’ OR Zambia OR Zimbabwe)
2	Scopus	(‘cell phone’ OR ‘mobile phone’ OR telephone OR ‘digital phone’ OR ‘mobile app’ OR ‘mobile application’ OR digital OR electronic OR automatic OR cyber OR automated OR programmed OR online OR multimedia OR ‘web-based’ OR robotic OR interactive OR virtual OR ‘digital technology’ OR ‘information technology’ OR technology OR tech OR ‘digital technologies’ OR e-health OR eHealth OR m-health OR mHealth OR ‘mobile health’ OR ‘mobile health applications’ OR ‘mobile apps’ OR ‘health apps’ OR ‘health applications’ OR ‘health tech’ OR telehealth OR telemedicine OR telecare OR ‘online health’ OR ‘web-based health’ OR ‘internet-based health’ OR ‘digital tools’ OR ‘digital platforms’ OR ‘digital solutions’ OR ‘electronic health’ OR ‘electronic health records’ OR ‘smartphone health’ OR ‘smartphone apps’ OR ‘wearable technology’ OR wearables OR ‘virtual health’ OR ‘virtual care’ OR ‘big data’ OR ‘artificial intelligence’ OR ‘real-time’ OR video OR applications OR apps OR ‘smart phone’) AND (oral health OR dental care OR dental health OR dental hygiene OR oral care OR odontic) AND (Angola OR Benin OR Botswana OR ‘Burkina Faso’ OR Burundi OR Cameroon OR ‘Cape Verde’ OR ‘Central African Republic’ OR Chad OR Comoros OR ‘Congo (Brazzaville)’ OR ‘Congo (Democratic Republic)’ OR ‘Côte d’Ivoire’ OR Djibouti OR ‘Equatorial Guinea’ OR Eritrea OR Ethiopia OR Gabon OR ‘The Gambia’ OR Ghana OR Guinea OR ‘Guinea-Bissau’ OR Kenya OR Lesotho OR Liberia OR Madagascar OR Malawi OR Mali OR Mauritania OR Mauritius OR Mozambique OR Namibia OR Niger OR Nigeria OR Réunion OR Rwanda OR ‘Sao Tome and Principe’ OR Senegal OR Seychelles OR ‘Sierra Leone’ OR Somalia OR ‘South Africa’ OR Sudan OR Swaziland OR Tanzania OR Togo OR Uganda OR ‘Western Sahara’ OR Zambia OR Zimbabwe)
3	Web of Science	TS = (‘cell phone’ OR ‘mobile phone’ OR telephone OR ‘digital phone’ OR ‘mobile app’ OR ‘mobile application’ OR digital OR electronic OR automatic OR cyber OR automated OR programmed OR online OR multimedia OR ‘web-based’ OR robotic OR interactive OR virtual OR ‘digital technology’ OR ‘information technology’ OR technology OR tech OR ‘digital technologies’ OR e-health OR eHealth OR m-health OR mHealth OR ‘mobile health’ OR ‘mobile health applications’ OR ‘mobile apps’ OR ‘health apps’ OR ‘health applications’ OR ‘health tech’ OR telehealth OR telemedicine OR telecare OR ‘online health’ OR ‘web-based health’ OR ‘internet-based health’ OR ‘digital tools’ OR ‘digital platforms’ OR ‘digital solutions’ OR ‘electronic health’ OR ‘electronic health records’ OR ‘smartphone health’ OR ‘smartphone apps’ OR ‘wearable technology’ OR wearables OR ‘virtual health’ OR ‘virtual care’ OR ‘big data’ OR ‘artificial intelligence’ OR ‘real-time’ OR video OR applications OR apps OR ‘smart phone’) AND (oral health OR dental care OR dental health OR dental hygiene OR oral care OR odontic) AND TS = (Angola OR Benin OR Botswana OR ‘Burkina Faso’ OR Burundi OR Cameroon OR ‘Cape Verde’ OR ‘Central African Republic’ OR Chad OR Comoros OR ‘Congo (Brazzaville)’ OR ‘Congo (Democratic Republic)’ OR ‘Côte d’Ivoire’ OR Djibouti OR ‘Equatorial Guinea’ OR Eritrea OR Ethiopia OR Gabon OR ‘The Gambia’ OR Ghana OR Guinea OR ‘Guinea-Bissau’ OR Kenya OR Lesotho OR Liberia OR Madagascar OR Malawi OR Mali OR Mauritania OR Mauritius OR Mozambique OR Namibia OR Niger OR Nigeria OR Réunion OR Rwanda OR ‘Sao Tome and Principe’ OR Senegal OR Seychelles OR ‘Sierra Leone’ OR Somalia OR ‘South Africa’ OR Sudan OR Swaziland OR Tanzania OR Togo OR Uganda OR ‘Western Sahara’ OR Zambia OR Zimbabwe)
4	Google Scholar	(‘cell phone’ OR ‘mobile phone’ OR telephone OR ‘digital phone’ OR ‘mobile app’ OR ‘mobile application’ OR digital OR electronic OR automatic OR cyber OR automated OR programmed OR online OR multimedia OR ‘web-based’ OR robotic OR interactive OR virtual OR ‘digital technology’ OR ‘information technology’ OR technology OR tech OR ‘digital technologies’ OR e-health OR eHealth OR m-health OR mHealth OR ‘mobile health’ OR ‘mobile health applications’)
